# Dynamic Graph Convolutional Network with Dilated Convolution for Epilepsy Seizure Detection

**DOI:** 10.3390/bioengineering12080832

**Published:** 2025-07-31

**Authors:** Xiaoxiao Zhang, Chenyun Dai, Yao Guo

**Affiliations:** 1 Department of Neurosurgery, Ruijin Hospital, Shanghai Jiao Tong University School of Medicine, Shanghai 200025, China; 13636399065@163.com; 2 School of Biomedical Engineering, Shanghai Jiao Tong University, Shanghai 200230, China; chenyundai@sjtu.edu.cn

**Keywords:** seizure detection, graph convolutional networks, EEG

## Abstract

The electroencephalogram (EEG), widely used for measuring the brain’s electrophysiological activity, has been extensively applied in the automatic detection of epileptic seizures. However, several challenges remain unaddressed in prior studies on automated seizure detection: (1) Methods based on CNN and LSTM assume that EEG signals follow a Euclidean structure; (2) Algorithms leveraging graph convolutional networks rely on adjacency matrices constructed with fixed edge weights or predefined connection rules. To address these limitations, we propose a novel algorithm: Dynamic Graph Convolutional Network with Dilated Convolution (DGDCN). By leveraging a spatiotemporal attention mechanism, the proposed model dynamically constructs a task-specific adjacency matrix, which guides the graph convolutional network (GCN) in capturing localized spatial and temporal dependencies among adjacent nodes. Furthermore, a dilated convolutional module is incorporated to expand the receptive field, thereby enabling the model to capture long-range temporal dependencies more effectively. The proposed seizure detection system is evaluated on the TUSZ dataset, achieving AUC values of 88.7% and 90.4% on 12-s and 60-s segments, respectively, demonstrating competitive performance compared to current state-of-the-art methods.

## 1. Introduction

Epilepsy is a neurological condition that impacts over 50 million people worldwide [1]. The analysis of brain waves, captured through electroencephalography (EEG), plays a vital role in diagnosing epileptic seizures [2]. However, manually labeling seizure events is a time-consuming and labor-intensive task for medical professionals. This has led to the pressing need for the development of automated seizure detection methods utilizing deep learning techniques. In parallel with advances in deep learning, initiatives like the Seizure Community Open-Source Research Evaluation (SzCORE) framework have been proposed to establish standardized benchmarks and reproducible evaluation pipelines for EEG-based seizure detection algorithms [3]. Such frameworks promote fair comparisons across different methods and enhance their clinical applicability by ensuring that algorithms are validated on common standards. By using SzCORE or similar evaluation platforms, researchers can more objectively assess performance, which is crucial for translating these algorithms into reliable clinical tools.

In recent years, traditional machine learning algorithms such as convolutional neural network (CNN) [4], recurrent neural network (RNN) [5], and long short-term memory (LSTM) [6] have achieved remarkable success in automated EEG event detection. However, with rapid advancements in deep learning technologies, integrating cutting-edge methods into existing frameworks holds the potential to significantly enhance the accuracy of EEG event detection. Improvements can be pursued in several critical areas. For CNN-based approaches, there are notable limitations. First, CNN models primarily focus on capturing short-term EEG features, often failing to account for the long-term dependencies that are intrinsic to EEG signals. Given that EEG data exhibits strong long-term correlations, it is crucial to explore more advanced models capable of effectively extracting these dependencies. Second, the spatial configuration of EEG electrodes provides valuable insights, but CNN are inherently limited in capturing this information due to their reliance on a Euclidean framework. This approach does not align with the non-Euclidean, irregular arrangement of EEG electrodes on the scalp. Similarly, RNN and LSTM also face significant challenges. First, they struggle to effectively capture relationships across different channels, which is critical in EEG analysis. Second, these models are computationally intensive to train and exhibit low efficiency when processing long sequences, further limiting their applicability to EEG event detection. Addressing these limitations is essential for advancing EEG event detection models. Graph Neural Networks (GNN), in particular, offer a promising solution by effectively overcoming the constraints of CNN, RNN, and LSTM.

In the early stages of research, GNN primarily focused on modeling spatial information. Kipf et al. proposed a method that directly operates on graph structures and performs convolutions on graph data, known as graph convolutional networks (GCN) [7]. A notable feature of this network is its first-order property, enabling a single-layer GCN to process first-order neighbor information in a graph, while multi-layer GCN models can further integrate K-order neighbor information. However, classical GCNs are more suitable for static graph data, where nodes and edges remain fixed and do not change over time. In 2018, the DCRNN model was proposed, leveraging an RNN architecture and bidirectional random walks to capture both temporal and spatial correlations [8]. Subsequently, Tang et al. utilized the encoder component of the DCRNN model to achieve state-of-the-art results in cross-subject generalized epileptic seizure detection [9]. However, this model also exhibits inherent limitations when applied to seizure detection. Firstly, the RNN architecture suffers from unstable gradients and high memory requirements. More importantly, the static adjacency matrix in the model relies on fixed edge weights or connection rules. When noise or outliers are present in the data, this static dependency can amplify the impact of such noise, preventing the model from dynamically adapting to changes in the input data. Graph Attention Networks (GAT) were introduced to address the rigidity of fixed adjacency matrices by learning attention weights for each node’s neighbors [10]. However, GAT integrates attention implicitly during message passing and does not explicitly decouple spatial and temporal correlations—an important property for biomedical signals such as EEG. Moreover, GAT requires dense attention computation at each layer, which incurs significant computational overhead and limits scalability in large-scale or high-density EEG data. To address the aforementioned challenges, we propose a Dynamic Graph Convolutional Network with Dilated Convolution (DGDCN). The main contributions of our work can be summarized as follows:

- The proposed model utilizes a spatiotemporal attention mechanism to adaptively generate a task-specific adjacency matrix, which in turn guides GCN to better model localized spatial and temporal relationships among neighboring nodes. Additionally, a dilated convolutional module is integrated to broaden the receptive field, enabling the network to more effectively capture long-term temporal dependencies.
- The effectiveness of the proposed DGDCN has been thoroughly evaluated using the TUSZ database, where it consistently demonstrates competitive performance compared to other state-of-the-art approaches. This exceptional performance underscores the model’s capability to handle complex EEG data, making it a valuable tool for advancing both research and practical applications in the field.

## 2. Methods

**Figure 1:**
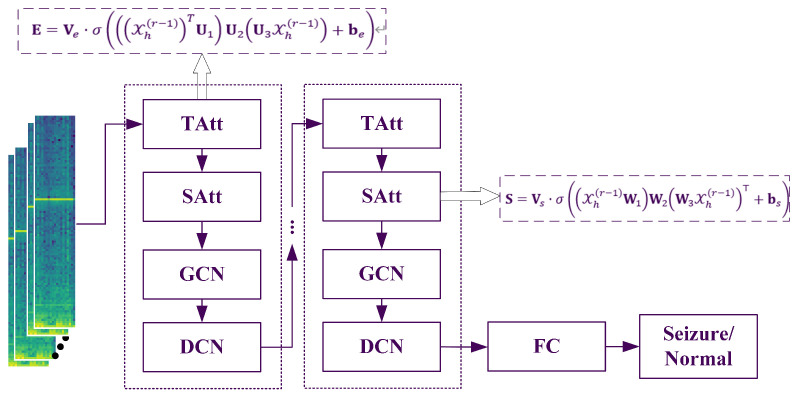
The flowchart of the proposed method (TAtt denotes the Time Attention module, SAtt represents the Space Attention module, and FC stands for the fully connected layer).

The proposed model incorporates a dynamic attention mechanism to effectively capture the evolving spatiotemporal correlations in epilepsy recognition networks. In parallel, a spatiotemporal convolution module is specifically designed to extract both spatial and temporal dependencies from neighboring nodes. Furthermore, the model employs a dilated convolutional network to stack multiple spatiotemporal blocks, enabling the extraction of more comprehensive and large-scale dynamic spatiotemporal correlations. The entire flowchart is illustrated in Figure 1.

### 2.1. Temporal Attention

In the temporal dimension, there are correlations between the EEG states of preceding and succeeding time segments. Apart from the epileptic seizure state, the correlations in EEG induced by noise also exhibit slight differences. Similarly, an attention mechanism can be used to adaptively assign different levels of importance to the data [11].

(1) $$E=V_{e}\cdot \sigma \left(\left({\left(X_{h}^{\left(r-1\right)}\right)}^{T}U_{1}\right)U_{2}\left(U_{3}X_{h}^{\left(r-1\right)}\right)+b_{e}\right)$$

(2) $$E_{i,j}^{\prime }=\frac{\exp\left(E_{i,j}\right)}{\sum_{j=1}^{T_{r-1}}\exp\left(E_{i,j}\right)}$$

Here, $V_{e},b_{e}\in R^{T_{r-1}\times T_{r-1}},U_{1}\in R^{N},U_{2}\in R^{C_{r-1}\times N},U_{3}\in R^{C_{r-1}}$ are learnable parameters. The learnable matrices $U_{1}$, $U_{2}$, and $U_{3}$ serve to project and combine spatial and feature information across time steps. Specifically, $U_{1}\in R^{N}$ aggregates node-level activations, $U_{2}\in R^{C\times N}$ integrates spatial and channel information, and $U_{3}\in R^{C}$ performs feature weighting across channels. These projections enable the attention mechanism to compute temporal correlations that reflect variations in EEG dynamics across different time steps. The temporal correlation matrix *E* is determined by the time-varying input data. Each element $E_{ij}$ represents the strength of the correlation between times *i* and *j*. Finally, *E* is normalized using a softmax function. The normalized temporal attention matrix is directly applied to the input, resulting in ${\hat{X}}_{h}^{\left(r-1\right)}=\left({\hat{X}}_{1},{\hat{X}}_{2},\ldots ,{\hat{X}}_{T_{r-1}}\right)=\left(X_{1},X_{2},\ldots ,X_{T_{r-1}}\right)$, where $E^{\prime }\in R^{N\times C_{r-1}\times T_{r-1}}$ can combine the correlation information to dynamically adjust the input.

### 2.2. Spatial Attention

In the spatial dimension, EEG signals from different channels influence one another, and this interaction is highly dynamic. An attention mechanism is employed here to adaptively capture the dynamic correlations between nodes in the spatial dimension. The spatial attention is computed as follows:

Let $X_{h}^{\left(r-1\right)}=\left(X_{1},X_{2},\ldots ,X_{T_{r-1}}\right)\in R^{N\times C_{r-1}\times T_{r-1}}$ represent the input of the *r*-th spatial block at the *r*-th layer, where *N* is the number of channels in the input data at layer *r*. When r=1, $C_{0}=F_{0}$, and $T_{r-1}$ is the length of the temporal dimension at the *r*-th layer. $V_{s},b_{s}\in R^{N\times N},W_{1}\in R^{T_{r-1}},W_{2}\in R^{C_{r-1}\times T_{r-1}},W_{3}\in R^{C_{r-1}}$ are learnable parameters, and σ denotes the activation function, here chosen as the sigmoid function. The attention matrix *S* is computed based on the dynamics of the input data at the current layer. Each element $S_{ij}$ in the matrix *S* indicates the correlation strength between EEG channels *n* and *m*. The softmax function is then applied to normalize *S*, ensuring that the attention weights across channels sum to 1. During subsequent convolutional operations, the normalized spatial attention matrix $S\in R^{N\times N}$ dynamically adjusts the influence between channels.

(3) $$S=V_{s}\cdot \sigma \left(\left(X_{h}^{\left(r-1\right)}W_{1}\right)W_{2}{\left(W_{3}X_{h}^{\left(r-1\right)}\right)}^{⊤}+b_{s}\right)$$

(4) $$S_{n,m}^{\prime }=\frac{\exp\left(S_{n,m}\right)}{\sum_{m=1}^{N}\exp\left(S_{n,m}\right)}$$

While conventional graph-based methods often enforce symmetry or sparsity constraints on the adjacency matrix, we do not explicitly apply such constraints to the spatial attention matrix S. The learned $S\in R^{N\times N}$ is generally asymmetric, as it is computed in a data-driven manner without enforcing $S=S^{⊤}$. This design allows the model to capture directed and potentially asymmetric dependencies between EEG channels, which may reflect meaningful patterns of information flow in neural systems. Furthermore, we do not apply thresholding or binarization to sparsify S. In the context of EEG, each channel is part of a functionally integrated system, and even weak correlations between electrode pairs may carry subtle but informative neural signals. By retaining all pairwise weights, our model remains sensitive to both strong and weak dependencies, which may be crucial for decoding complex brain dynamics.

### 2.3. Spatiotemporal Convolution Based on GCN

The spatiotemporal attention module efficiently enables the network to adaptively focus on more valuable channel and temporal information. The input adjusted by the attention mechanism is fed into the spatiotemporal convolution module. The proposed spatiotemporal convolution module consists of graph convolutions in the spatial dimension, capturing spatial dependencies from neighboring nodes, and convolutions along the temporal dimension, leveraging temporal dependencies from adjacent time steps.

The Laplacian matrix of a graph is defined as L=D−A, and it can further be normalized as $L=I_{N}-D^{-\frac{1}{2}}AD^{-\frac{1}{2}}$, where *A* is the adjacency matrix, $I_{N}$ is the identity matrix, and $D\in R^{N\times N}$ is the degree matrix, a diagonal matrix formed by the node degrees $D_{ii}=\sum_{j}A_{ij}$. The eigenvalue decomposition of the Laplacian matrix is $L=U\Lambda U^{T}$, where $\Lambda =\mathrm{diag}(\lambda_{0},\ldots ,\lambda_{N-1})$ is a diagonal matrix of eigenvalues, and *U* is the Fourier basis of the graph. Taking the EEG signal at a specific time as an example, the signal over the entire graph is $x\in R^{N}$, and its graph Fourier transform is defined as $\hat{x}=U^{T}x$. Based on the properties of the Laplacian matrix, *U* is an orthogonal matrix, so the corresponding inverse Fourier transform is $x=U\hat{x}$. Graph convolution is implemented using a linear operator that is diagonalized in the Fourier domain, effectively replacing classical convolution operators. Based on this, the signal *x* on graph *G* is filtered using the kernel $g_{\theta }$ as follows:

(5) $$g_{0}{*}_{G}x=g_{\theta }\left(L\right)x=\sum_{k=0}^{K-1}\theta_{k}T_{k}\left(\bar{L}\right)x$$

The parameter $\theta \in R^{K}$ is the direction of the polynomial coefficients. $\tilde{L}=\frac{2}{\lambda_{\max}}L-I_{N}$, where $\lambda_{\max}$ is the largest eigenvalue of the Laplacian matrix. The recursive definition of the Chebyshev polynomial is $T_{k}\left(x\right)=2xT_{k-1}\left(x\right)-T_{k-2}\left(x\right)$, where $T_{0}\left(x\right)=1$ and $T_{1}\left(x\right)=x$. By using an approximation of the Chebyshev polynomial, this formulation allows extracting information from neighbors up to K−1 hops away for each node through the convolution kernel $g_{\theta }$. The graph convolution module applies a rectified linear unit (ReLU) as the final activation function, expressed as: $\mathrm{ReLU}(g_{\theta }*x)$.

To dynamically capture correlations between source and destination nodes, for each term of the Chebyshev polynomial, the spatial attention matrix $S\in R^{N\times N}$ is incorporated. The resulting matrix is $T_{k}\left(\tilde{L}\right)⊙S^{\prime }$, where ⊙ denotes the Hadamard product (element-wise multiplication). Hence, the graph convolution formula becomes:

(6) $$g_{\theta }*Gx=g_{\theta }\left(L\right)x=\sum_{k=0}^{K-1}\theta_{k}\left(T_{k}\left(\tilde{L}\right)⊙S^{\prime }\right)x$$

This definition is extended to multi-channel graph signals. For example, the input is $X_{h}^{\left(r-1\right)}=\left(X_{1},X_{2},\ldots ,X_{T_{r-1}}\right)\in R^{N\times C_{r-1}\times T_{r-1}}$, where each node’s feature vector contains $C_{r-1}$ channels at each time step. For the current graph *G*, convolution is applied using $g_{\theta }*Gx$, where $\theta \in R^{C_{r-1}\times C_{r}}$ represents the learnable parameters of the kernel. Consequently, each node updates its information based on its neighbors within 0 to K−1 hops.

### 2.4. Dilated Convolutional Network

A dilated convolution is a convolutional operation that introduces gaps (or dilations) between the kernel elements, allowing the network to aggregate information from a broader context without increasing the number of parameters [12,13]. Specifically, with a dilation rate of *d*, the convolutional kernel skips d−1 elements between each sampled position. This mechanism effectively expands the receptive field, enabling the model to capture long-range dependencies. In the context of graph-based temporal modeling, dilated convolutions are particularly useful for incorporating information from distant time steps while maintaining computational efficiency.

The graph convolution operation captures the neighboring information of each node in the spatial dimension of the graph, followed by an aggregation step in the temporal dimension. By combining the information from neighboring time segments, the node signals are updated. The operation in the most recent component is expressed as follows:

(7) $$X_{h}^{\left(r\right)}=\mathrm{ReLU}\left(\Phi *\left(\mathrm{ReLU}\left(g_{\theta }*X_{h}^{\left(r-1\right)}\right)\right)\right)$$

where * represents a spatial convolution operation, Φ contains the parameters of the convolution kernel, and its expansion rate is set to 2. The activation function of the convolution layer is ReLU.

We note that the input and output dimensions remain consistent throughout the main components of our model. The temporal attention and spatial attention + GCN modules are designed to preserve the original tensor shape, as they perform feature reweighting and aggregation without altering the spatial or temporal resolution. Furthermore, the DCN module is applied with appropriate padding to ensure that the temporal dimension remains unchanged. These design choices ensure that each module can be stacked multiple times without introducing dimensional inconsistencies, thereby simplifying model design and improving scalability.

In summary, the spatiotemporal convolution module effectively captures the spatiotemporal features of the data. With the spatiotemporal attention module, GCN and DCN form spatiotemporal blocks. Stacking multiple spatiotemporal blocks allows the model to capture dynamic spatiotemporal correlations at a broader scale. Finally, a fully connected layer is added, with ReLU serving as the activation function for the final fully connected layer.

## 3. Experimental Setup

### 3.1. Experimental Data

This study utilizes version 2.0.1 of the TUSZ (Temple University Seizure Corpus) database for experimental validation. Provided by the Temple University Neural Engineering Data Consortium, TUSZ is currently the largest publicly available epilepsy database [14]. The EEG recordings in this database were primarily collected between 2002 and 2013 (and subsequent years) at Temple University. Notably, TUSZ is one of the few epilepsy databases that continues to receive annual updates. The database contains data from a total of 675 patients, amounting to 5,312,996 s of recordings and 81 GB of data. It includes 7377 EDF files, data from 287 seizure patients, and 4,029 seizure events. All recordings were collected using 22-channel EEG equipment, with key channels including FP1-F7, F7-T3, T3-T5, T5-O1, FP2-F8, F8-T4, T4-T6, T6-O2, A1-T3, T3-C3, C3-CZ, CZ-C4, C4-T4, T4-A2, FP1-F3, F3-C3, C3-P3, P3-O1, FP2-F4, F4-C4, C4-P4, and P4-O2. To facilitate usage, the database is divided into three subsets: training (train), development (dev), and evaluation (eval). The total recording durations for these subsets are 3,277,229 s, 467,795 s, and 1,567,972 s, respectively. The proportion of seizure durations in these subsets is 5.34%, 5.09%, and 5.82%, respectively, and each subset contains data from distinct, non-overlapping patients. Typically, researchers use the training set for model training, the development set for hyperparameter tuning, and the evaluation set for assessing final model performance. One challenge of the TUSZ database is its high data diversity and significant noise, often leading to lower validation performance compared to other databases. However, since its subsets are pre-defined and independent of validation methods (e.g., leave-one-out or k-fold cross-validation), many researchers use TUSZ as a benchmark dataset for evaluating the performance of deep learning models. To support reproducibility and future research, we publicly release the implementation code at https://github.com/Open-EMG/DGDCN.git (accessed on 31 July 2025).

### 3.2. Data Preprocessing

To ensure consistency, EEG signals from the TUSZ, originally sampled at varying frequencies, are resampled to a uniform frequency of 200 Hz [14]. Given that seizures correlate with brain electrical activity in specific frequency bands, we preprocess the raw EEG signals by transforming them into the frequency domain [15]. Following prior studies, we compute the log-amplitudes of the fast Fourier transform (FFT) of the raw EEG data [9]. EEG recordings were divided into non-overlapping segments of 12 and 60 s. A segment was labeled as ictal if it contained at least one second of seizure activity, following a labeling strategy similar to [9]. Using both short (12 s) and long (60 s) segments allows us to evaluate the model under different temporal granularities, and captures both fine-grained ictal events and broader contextual patterns around seizure episodes. Specifically: (1) A sliding window of *t* seconds is applied over the EEG clips without overlap, where *t* matches the time step; (2) The FFT is computed for each *t*-s window using the “fft” function in the Scipy Python library, retaining the log-amplitudes of the non-negative frequency components as described in prior studies; (3) Finally, the EEG clips are z-normalized with respect to the mean and standard deviation of the training dataset. After preprocessing, each EEG clip is represented as $X\in R^{N\times C\times T}$, where T=12 (or T=60 for longer clips) represents the clip length, N=22 denotes the number of EEG channels/electrodes, and C=100 corresponds to the feature dimension derived from the Fourier transform. Each node in the graph corresponds to an EEG electrode, and the features associated with each node are frequency-domain representations of the EEG signals obtained through a Fourier transform. Specifically, for each time step, a 100-dimensional spectral feature vector is extracted for each of the 22 channels, capturing key frequency characteristics such as the power distribution across different bands. These representations are essential for modeling seizure-related dynamics. As a result, each node is associated with a sequence of spectral features over time, forming the structured input tensor $X\in R^{N\times C\times T}$ used for subsequent spatiotemporal modeling.

### 3.3. Model Training

All models, including the baseline models, were implemented using the PyTorch library and trained on an NVIDIA GeForce RTX 4080 GPU with the Adam optimizer. The initial parameters were randomly initialized. The hyperparameter settings for DGDCN are as follows: the initial learning rate was set to 1e-4, the batch size was 20, and the number of terms for the Chebyshev polynomial was 3. All graph convolution layers used 64 convolutional kernels, and all temporal convolution layers also used 64 convolutional kernels. All models were trained using the cosine annealing learning rate scheduling strategy in PyTorch [16]. This strategy gradually updates the learning rate following a cosine waveform decay cycle. Additionally, in all experiments, early stopping was applied if the validation loss did not decrease for five consecutive iterations. To mitigate the impact of class imbalance between resting and ictal EEG segments, we applied two EEG-specific data augmentation techniques during training: (1) amplitude scaling within the range [0.8, 1.2], and (2) random reflection across the scalp midline.

### 3.4. Evaluating Indicator

For evaluation, in addition to accuracy, we report AUC, sensitivity, and specificity, which are more robust to imbalanced data distributions. AUC reflects the model’s ability to discriminate between classes across all decision thresholds, making it particularly suitable for seizure detection tasks with skewed class ratios. Accuracy measures the proportion of correctly classified samples to the total number of samples, reflecting the overall correctness of the model’s predictions.

(8) $$\mathrm{ACC}=\frac{\mathrm{TP}+\mathrm{TN}}{\mathrm{TP}+\mathrm{TN}+\mathrm{FP}+\mathrm{FN}}$$

where True Positive (TP) denotes a seizure-labeled EEG clip that is correctly predicted as a seizure; True Negative (TN) refers to a non-seizure EEG clip that is correctly predicted as non-seizure; False Positive (FP) represents a non-seizure EEG clip that is incorrectly classified as seizure; and False Negative (FN) indicates a seizure-labeled EEG clip that is incorrectly predicted as non-seizure. These definitions provide the basis for computing additional performance metrics such as sensitivity, specificity, and AUC.

Sensitivity indicates the model’s ability to identify positive (Seizure) cases, i.e., the proportion of actual positive samples correctly predicted as positive. It is particularly crucial in tasks where minimizing false negatives is a priority.

(9) $$\mathrm{Sensitivity}=\frac{\mathrm{TP}}{\mathrm{TP}+\mathrm{FN}}$$

Specificity evaluates the model’s ability to identify negative (normal) cases, i.e., the proportion of actual negative samples correctly predicted as negative. It is particularly useful in reducing false positives and complementing sensitivity.

(10) $$\mathrm{Specificity}=\frac{\mathrm{TN}}{\mathrm{TN}+\mathrm{FP}}$$

AUC represents the area under the Receiver Operating Characteristic (ROC) curve and serves as a comprehensive metric to evaluate the model’s ability to distinguish between positive and negative samples. An AUC value closer to 1 indicates a strong discriminative ability, while an AUC value of 0.5 suggests the model performs no better than random guessing. Unlike accuracy, AUC is robust to class imbalance.

Since the task of epilepsy detection involves imbalanced data and clinicians are primarily concerned with ensuring that all actual seizures are accurately identified, SEN and AUC are the most critical evaluation metrics for this task.

### 3.5. Benchmark Methods

To evaluate the effectiveness of the proposed model compared to other models, four baselines, including LSTM, CNN, EEGNET [17], and DCRNN [9], were selected. All baselines used the same data and preprocessing methods as DGDCN and were evaluated using the same metrics.

## 4. Results

This study conducted a comprehensive comparison between the proposed model and four benchmark methods: LSTM, CNN, EEGNET, and DCRNN. Table 1 presents the final performance evaluation results, covering accuracy, sensitivity, specificity, and AUC. From the table, it is evident that the DGDCN model consistently achieves competitive performance across all metrics compared to the benchmark methods. Notably, when processing 12-s data segments, DGDCN demonstrates superior performance across all evaluation criteria. Even when handling 60-s data segments, the model maintains its advantage in the two most critical metrics—sensitivity and AUC.

For 12-s input segments, the proposed DGDCN model achieved an accuracy of 79.7%, sensitivity of 84.8%, specificity of 78.6%, and AUC of 88.7%. For 60-s input segments, the model achieved an accuracy of 73.5%, sensitivity of 94.3%, specificity of 68.7%, and AUC of 90.4%.

To evaluate the importance of each module, ablation experiments were conducted by removing individual modules from the model and testing its performance. The results of these experiments are summarized in Table 2.

1. Removal of the Temporal Attention Module: This corresponds to ignoring the correlations between different time segments. The results show that incorporating this module enhances both sensitivity and AUC. For 12-s data, sensitivity and AUC improved by 1.7% and 0.3%, respectively. Similarly, for 60-s data, these metrics increased by 1% and 0.2%, respectively.

2. Removal of the Spatial Attention Module: This involves disregarding the correlations between different spatial regions. The results indicate that removing this module leads to significant declines in sensitivity and specificity. For 12-s data, sensitivity dropped by 4.2%. For 60-s data, sensitivity and AUC decreased by 4.4% and 1.8%, respectively.

3. Removal of the Entire GCN Component: The results demonstrate that eliminating the GCN module results in a substantial reduction in sensitivity and AUC. For 12-s data, sensitivity and AUC decreased by 3.1% and 3%, respectively. For 60-s data, these metrics dropped by 3.4% and 0.7%, respectively.

4. Removal of the Entire DCN Component: The results show that removing the DCN module reduces sensitivity and AUC. For 12-s data, these metrics dropped by 0.7% and 0.2%, respectively. However, for 60-s data, sensitivity and AUC declined more drastically, by 9.1% and 2.2%, respectively. Notably, the performance degradation for 60-s data is more pronounced compared to 12-s data, further confirming that the dilated convolution within the DCN module enhances the model’s ability to process long sequence data effectively.

These results highlight the critical contributions of each module to the overall performance of the model, particularly in capturing spatiotemporal correlations and handling long-sequence data.

Further research explored the impact of the data augmentation module on the model’s performance, as detailed in Table 3. From the table, it can be observed that incorporating data augmentation enhances the model’s performance when processing long-sequence data. Without data augmentation, the sensitivity and AUC for 60-s data segments decrease notably by 5.7% and 1%, respectively. However, when the data augmentation module is applied, its effect on short-sequence data is less pronounced. While sensitivity improves slightly, the overall AUC metric shows a marginal decline.

## 5. Discussion

The results presented in this study demonstrate the superior performance of the proposed DGDCN model over benchmark methods such as LSTM, CNN, EEGNET, and DCRNN. DGDCN consistently demonstrates favorable results across all evaluation metrics, especially in sensitivity and AUC. These findings indicate the model’s capability to effectively handle the complexities of EEG data, especially in imbalanced classification tasks such as epileptic seizure detection, where reducing false negatives (i.e., improving sensitivity) is crucial.

One of the key findings is the significant advantage of DGDCN in processing both short (12-s) and long (60-s) EEG segments. While CNN and EEGNET perform poorly across different segment lengths, likely due to their inability to capture long-term temporal dependencies, LSTM shows relatively stable AUC but struggles with structural inconsistencies in EEG signals. The ability of DGDCN to leverage graph-based architectures to model non-Euclidean relationships allows it to capture spatiotemporal dependencies more effectively. The comparison between DCRNN and DGDCN highlights the latter’s improved performance, particularly in handling long-sequence data, with notable increases in AUC.

Ablation experiments further validate the contributions of individual components in DGDCN. The removal of the TAtt leads to decreased sensitivity and AUC, confirming its role in enhancing temporal correlations. The SAtt also proves essential, as its removal lowers sensitivity and specificity, indicating its importance in capturing spatial dependencies in EEG signals. Eliminating the GCN module results in a notable reduction in both sensitivity and AUC, underscoring the necessity of graph-based modeling. Additionally, the removal of the DCN module causes the most substantial performance drop, particularly for 60-s data, reaffirming its role in handling long-sequence data effectively.

These findings emphasize that the DGDCN model successfully integrates dynamic spatiotemporal information using graph-based and dilated convolutional mechanisms, making it a robust solution for EEG-based seizure detection. The superior performance of DGDCN in long-sequence data processing suggests its potential applicability in real-world clinical settings, where EEG signals often vary in duration.

Further experiments also indicate that data augmentation enhances the model’s performance in processing long-sequence EEG data. Without augmentation, sensitivity and AUC decrease notably, emphasizing the importance of training data diversity in improving model robustness. Interestingly, although data augmentation significantly improved sensitivity in both the 12-s and 60-s settings, it resulted in a slight reduction in AUC for the 12-s segments. This may be due to the fact that shorter EEG windows contain less temporal variability, making them more sensitive to distortions introduced by augmentation. While such augmentation enhances the model’s ability to detect seizures overall, it may also increase uncertainty for borderline cases, thereby destabilizing the decision boundary and slightly reducing AUC.

Overall, the results confirm that DGDCN effectively addresses the challenges of spatiotemporal EEG classification, particularly in seizure detection. The model’s performance across various segment lengths, along with its use of key attention-based components, demonstrates promising potential for clinical deployment, showing results that are comparable to or better than traditional methods. Future work may focus on extending this approach to other neurological disorders and further optimizing its real-time applicability in medical settings.

### Limitation

Recent evaluations by Koren et al. and Reus et al. have systematically assessed commercial seizure detection systems in real-world epilepsy monitoring unit settings [18,19]. While these tools demonstrate reasonable sensitivity and assist in seizure identification, their overall clinical reliability remains limited, particularly due to high false alarm rates, missed events, and poor robustness in noisy clinical environments. These findings underscore the importance of developing detection systems that are not only accurate on public datasets but also clinically viable in deployment scenarios.

Despite the promising performance of our proposed method on benchmark datasets, several limitations should be acknowledged. First, our current evaluation does not consider critical deployment-related factors such as detection latency, computational efficiency, and the ability to process continuous streaming EEG signals. Second, the system has not yet been validated in realistic clinical settings or integrated into hospital workflows, which poses challenges for practical adoption.

To address these limitations, future work should incorporate standardized benchmarking frameworks such as SzCORE. This platform offers unified evaluation protocols across multiple datasets and patient populations, enabling more objective comparisons and improving reproducibility. Integrating SzCORE could help verify our method’s generalizability under real-world conditions and facilitate its translation into clinical use.

Although our study is based on multi-channel EEG data, the proposed spatial-temporal attention framework is inherently flexible and can be adapted to settings with varying electrode layouts. First, the spatial attention matrix is learned from the input, which allows it to naturally scale with different channel configurations. Second, the dynamic graph construction mechanism enables the model to capture inter-channel dependencies even under sparse or individualized layouts. These properties support the potential clinical use of our method in reduced-channel EEG systems. Moreover, if the framework is combined with an internal electrode selection strategy, such as attention-based ranking or sparsity-regularized learning as proposed in related studies [20,21,22,23], it may not only reduce computational complexity but also enhance performance by concentrating on the most informative channels.

Lastly, while this study focuses on data-driven spatiotemporal modeling of EEG signals, true clinical validation also requires alignment with anatomical and electro-clinical information. For example, although the TUSZ dataset is widely used, it lacks metadata such as seizure onset zones or detailed semiology. Future research should incorporate richer clinical annotations to support more comprehensive and translationally meaningful validation.

## 6. Conclusions

In conclusion, this study addresses key limitations in automated seizure detection by introducing the Dynamic Graph Convolutional Network with Dilated Convolution, which effectively captures dynamic spatiotemporal correlations through a novel attention mechanism and adaptive adjacency matrix construction. The integration of a spatiotemporal convolution module and dilated convolutional network enables the extraction of broader temporal and spatial dependencies, leading to significant improvements in seizure detection performance. The proposed method achieves superior AUC scores of 88.7% and 90.4% on 12-s and 60-s EEG segments, respectively, on the TUSZ dataset, demonstrating its potential as a robust and advanced solution for epileptic seizure detection.

## Figures and Tables

**Table 1:** Comparison of average performance for different methods with segment lengths of 12 and 60 s.

Method	Length (s)	Accuracy (%)	Sensitivity(%)	Specficity (%)	AUC (%)
LSTM	12	76.6	80.4	75.7	87.0
	60	81.1	77.7	81.8	87.0
CNN	12	75.1	83.6	73.3	85.1
	60	77.6	67.1	80.0	82.3
EEGNET	12	76.3	80.3	75.5	83.5
	60	52.8	35.3	45.3	74.6
DCRNN	12	77.3	76.4	77.5	86.7
	60	79.5	82.6	78.6	88.4
This Work	12	79.7	84.8	76.6	88.7
	60	73.5	94.3	68.7	90.4

**Table 2:** Ablation Experiments.

Removed Module	12 s		60 s	
	**Sensitivity (%)**	**AUC (%)**	**Sensitivity (%)**	**AUC (%)**
TAtt	77.2	88.4	93.3	90.2
SAtt	80.6	88.7	89.9	88.6
GCN	81.7	88.4	90.9	89.7
DCN	83.9	88.5	85.2	88.2
This Work	84.8	88.7	94.3	90.4

**Table 3:** Impact of Data Augmentation on Model Performance.

Removed Module	12 s		60 s	
	**Sensitivity (%)**	**AUC (%)**	**Sensitivity(%)**	**AUC (%)**
w/o DA	81.3	89.3	88.6	89.4
This Work	84.8	88.7	94.3	90.4

## Data Availability

The Temple University Hospital EEG (TUH EEG) Corpus used in this study is publicly available at https://isip.piconepress.com/projects/nedc/html/tuh_eeg/ (accessed on 18 July 2025).
